# Meropenem/vaborbactam activity against carbapenem-resistant *Klebsiella pneumoniae* from catheter-related bloodstream infections

**DOI:** 10.3389/fcimb.2025.1616353

**Published:** 2025-07-31

**Authors:** Francesca Sivori, Massimo Francalancia, Mauro Truglio, Ilaria Cavallo, Carmelina Pronesti, Giorgia Fabrizio, Ilaria Celesti, Andrea Cazzani, Lorenzo Furzi, Fulvia Pimpinelli, Enea Gino Di Domenico

**Affiliations:** ^1^ Microbiology and Virology, San Gallicano Dermatological Institute IRCCS, Rome, Italy; ^2^ Hospital Infection Control Committee, Istituti Fisioterapici Ospitalieri (IFO), Rome, Italy; ^3^ Department of Biology and Biotechnology “C. Darwin”, Sapienza University of Rome, Rome, Italy

**Keywords:** carbapenem-resistant *Klebsiella pneumoniae*, multidrug-resistant plasmid, meropenem/vaborbactam, catheter-related bloodstream infections, biofilm-associated infections, surgical site infection

## Abstract

**Introduction:**

Carbapenem-resistant *Klebsiella pneumoniae* (CRKP) poses a significant threat in oncology settings due to its multidrug resistance and ability to form biofilms on indwelling medical devices.

**Methods:**

This study investigated the *in vitro* and *in vivo* activity of meropenem/vaborbactam (MEV) against two CRKP isolates recovered from catheter-related bloodstream infections in patients undergoing orthopedic oncologic surgery.

**Results:**

Whole-genome sequencing identified the isolates as ST101 and ST307, harboring resistance determinants including *bla_KPC-3_* and *bla_OXA-1_*, distributed across IncFII and IncFIB plasmid replicons. Both isolates exhibited extensive resistance to β-lactams, aminoglycosides, and fluoroquinolones but remained susceptible to MEV. Phenotypic assays revealed enhanced biofilm formation and metabolic activity compared to the reference strain Kp ATCC 13883 in the absence of hypervirulence-associated genes. MEV demonstrated bactericidal activity against both planktonic and biofilm-associated cells, with minimum bactericidal concentration (MBC_90_) and minimum biofilm eradication concentration (MBEC_90_) values of 0.5/8 μg/ml for CRKP ST101, 0.12/8 μg/ml for CRKP ST307, and 0.25/8 μg/ml for the Kp ATCC 13883 strain. In the *Galleria mellonella* infection model, MEV significantly improved larval survival following the CRKP challenge.

**Discussion:**

These findings demonstrate that MEV exhibits activity against planktonic and biofilm-associated CRKP cells and highlight the need for further investigation in managing catheter-related bloodstream infections caused by multidrug-resistant *K. pneumoniae*.

## 1 Introduction

Carbapenem-resistant *Klebsiella pneumoniae* (CRKP) represents a critical threat in oncology settings, where immunosuppression, invasive procedures, and prolonged hospital stays increase the risk of life-threatening infections. Among the various carbapenemases, *Klebsiella pneumoniae* carbapenemase (KPC) enzymes are the most prevalent and clinically significant. Their plasmid-mediated dissemination severely limits treatment options and is associated with high morbidity and mortality rates (Murray et al., 2022).

Catheter-related bloodstream infections (CRBSIs) caused by multidrug-resistant *K. pneumoniae* are a growing concern in oncology, particularly among neutropenic and immunocompromised patients. Orthopedic oncology settings are particularly high-risk due to the complexity of surgical procedures and the frequent use of indwelling devices, such as central venous catheters (Zimmerli and Moser, 2012). These devices are prone to colonization by biofilm-forming CRKP, which exhibit increased antibiotic tolerance and immune clearance. Biofilm formation contributes to persistent and recurrent infections and has also been identified as a major virulence factor linked to higher mortality rates in this vulnerable population (Gominet et al., 2017; Di Domenico et al., 2021, 2022; Lendak et al., 2021).

Meropenem/vaborbactam (MEV), a novel combination of a carbapenem with a boronic acid β-lactamase inhibitor, has shown potent activity against KPC-producing strains by effectively protecting meropenem from enzymatic degradation (Jorgensen and Rybak, 2018). It has been approved for treating complicated infections caused by multidrug-resistant Gram-negative bacteria, including those involving the bloodstream. Several studies have demonstrated its *in vitro* efficacy against KPC-producing Enterobacterales, and recent clinical data support its use in real-world settings (Tiseo et al., 2024). Italy represents a region of high endemicity for CRKP, predominantly driven by KPC-type carbapenemases. MEV demonstrated consistent *in vitro* activity against KPC-producing *K. pneumoniae* bloodstream isolates, with reported susceptibility rates of 87% (Gaibani et al., 2021). More recent nationwide surveillance data indicate that MEV retains high efficacy, with susceptibility exceeding 95% among KPC producers. When compared with other novel β-lactam/β-lactamase inhibitor combinations, imipenem/relebactam exhibited the highest susceptibility rate (97%), followed by MEV and ceftazidime/avibactam (93.9%) (Bianco et al., 2025). Notably, resistance to MEV and imipenem/relebactam was associated with structural alterations in outer membrane porins, including truncation of OmpK35 and the ins135GD insertion in OmpK36.

This study investigates the efficacy of MEV against CRKP isolates recovered from CRBSI in an orthopedic oncology hospital. We assessed minimum bactericidal concentration (MBC) and minimum biofilm eradication concentrations (MBEC) and characterized relevant resistance and virulence determinants. Additionally, the *Galleria mellonella* infection model was employed to evaluate the therapeutic potential of MEV *in vivo*.

By integrating phenotypic susceptibility testing, genotypic profiling, and an experimental infection model, this work aims to provide insight into the effectiveness of MEV in managing CRKP infections in a clinically challenging setting.

## 2 Methods

### 2.1 Strain collection and ethical approval

We examined two clinical isolates of carbapenem-resistant *K. pneumoniae* (CRKP) obtained from hospital-acquired infections in an orthopedic oncology unit. Isolates were obtained from blood cultures collected via central venous access from each patient under sterile conditions and plated on complete and selective media. These strains, part of the Bacterial Strain Collection at the Microbiology and Virology Unit of the San Gallicano Dermatological Institute, Istituti Fisioterapici Ospitalieri (IFO), were collected between January and March 2020. All relevant clinical and microbiological data were recorded in an electronic database.

Catheter-related bloodstream infection (CRBSI) was diagnosed when blood cultures drawn from the catheter hub became positive at least two hours earlier than when peripheral blood cultures of equal volume were simultaneously collected, as measured by an automated blood culture system (Rickard et al., 2021).

This study received approval from the Central Ethics Committee I.R.C.C.S. Lazio (Prot. 5179—18.04.2023, N. 1860/23), and all procedures followed established ethical guidelines and local regulatory requirements.

### 2.2 Microbiological diagnosis and strain characterization

Carbapenem-resistant *K. pneumoniae* isolates were recovered from patients with hospital-acquired infections (Di Domenico et al., 2021) and cultured on McConkey (Becton Dickinson, Germany) and blood agar plates (Becton Dickinson, Germany). MALDI-TOF MS (Bruker Daltonik, Germany) was used for bacterial identification, which was subsequently confirmed by 16S rRNA gene sequencing. Antimicrobial susceptibility testing was performed using the BD Phoenix™ automated system (Becton Dickinson Diagnostic Systems, USA) NMIC-474 panel, while colistin susceptibility was determined by broth microdilution (Thermo Scientific, USA). Results were interpreted in accordance with the EUCAST clinical breakpoints (http://www.eucast.org/clinical_breakpoints). The Cepheid Xpert Carba-R assay, integrated with the GeneXpert device (Cepheid, USA), was used to preliminarily detect the presence of *bla_KPC_*, *bla_VIM_*, *bla_OXA-48_*, *bla_IMP_*, and *bla_NDM_* (Di Domenico et al., 2020). Lateral flow immunoassay was used to detect clinically relevant carbapenemases, specifically KPC, IMP, NDM, VIM, and OXA-48 (Coris BioConcept, Belgium). *K. pneumoniae* ATCC 13883 was included as a reference strain in all phenotypic tests to provide an internal standard for assay calibration.

### 2.3 Whole-genome analysis

According to the manufacturer’s protocol, genomic DNA was extracted using the QIAsymphony DSP Virus/Pathogen Kit (Qiagen, Germany). The DNA was sequenced using a hybrid approach combining Illumina MiSeq and Nanopore GridION technologies. Quality control of Illumina reads was performed using FastQC while Nanopore data were assessed with nf-core/nanoseq v3.1.0 (Ewels et al., 2020). Quality metrics were summarized using MultiQC (Ewels et al., 2016). Genome assembly was conducted using Bactopia v.3.2.0 (Petit and Read, 2020) with default options. The pipeline employed the Dragonflye assembler in “short_polish” mode, where long reads were first assembled and subsequently polished using short reads. The assembly process incorporated Medaka (Oxford Nanopore Technologies, 2020) for consensus correction of Nanopore reads and Polypolish (Wick and Holt, 2022) for polishing with Illumina data.

The assembled genomes were characterized using multiple bioinformatic tools. Plasmid replicons were identified using PlasmidFinder v2.1.6 (Carattoli et al., 2014). Antimicrobial resistance determinants, virulence factors, sequence typing (MLST), and capsular loci (KL) were analyzed using Kleborate v3.1.3 (Lam et al., 2021). Kaptive v0.7.3 was used to identify capsule (K-locus) and lipopolysaccharide (O-locus) loci (Lam et al., 2022).

Biosynthetic gene clusters were identified using antiSMASH v8.0 (bacterial version) (Blin et al., 2023). Additional antibiotic resistance gene detection was performed using the Comprehensive Antibiotic Resistance Database (CARD) v4.0 and the Resistance Gene Identifier (RGI) tool v6.0.3 (Alcock et al., 2023), applying “perfect” and “strict” matches with ≥97% identity threshold. Virulence gene profiling was conducted using Kleborate and ABRicate v1.0.1.

Phylogenetic analysis of chromosomes was performed using kSNP v4.0 (Gardner et al., 2015) to identify core single-nucleotide polymorphisms. The maximum likelihood tree generated by kSNP was selected for phylogenetic inference. Plasmid phylogeny was assessed using Mash v2.3 (Ondov et al., 2016) for sequence similarity estimation. Phylogenetic trees were visualized using Interactive Tree Of Life (iTOL) (Letunic and Bork, 2024). Circular representations of chromosomal features were generated using Proksee (Grant et al., 2023), highlighting antimicrobial resistance genes, biosynthetic gene clusters, and other relevant genomic features.

### 2.4 Determination of meropenem/vaborbactam minimum bactericidal concentration (MBC_90_)

The bactericidal activity of MEV was assessed using the broth microdilution method in cation-adjusted Mueller-Hinton broth (CAMHB), following standardized procedures. MEV was tested in a concentration range of 0.008–16 μg/ml for meropenem, combined with a fixed 8 μg/ml of vaborbactam. Bacterial inocula were prepared by suspending overnight cultures grown on MacConkey agar in 0.45% saline and adjusted to a turbidity equivalent to a 0.5 McFarland standard (approximately 10^8^ CFU/ml). The suspension was diluted to approximately 10^5^ CFU/ml, and 100 μl was added to each well of a sterile 96-well flat-bottom polystyrene microtiter plate containing 100 μl of CAMHB. After 20 h of incubation at 37°C, minimum inhibitory concentrations (MICs) were recorded as the lowest concentration inhibiting visible bacterial growth. These MIC values confirmed susceptibility to MEV (≤2/8 μg/ml) for both CRKP isolates and the reference strain, and were in full agreement with results obtained using the automated BD Phoenix™ system. For determination of MBC_90_, viable bacterial counts were determined by serial dilution and plating on MacConkey agar, and results were expressed as CFU/ml. The MBC_90_ was defined as the lowest antibiotic concentration, resulting in a ≥90% reduction in CFU/ml relative to the untreated control.

### 2.5 Determination of meropenem/vaborbactam minimum biofilm eradication concentration (MBEC_90_)

Biofilm eradication assays were performed as previously described, with modifications. Overnight cultures grown on MacConkey agar were suspended in 0.45% saline and adjusted to 0.5 McFarland standard. The suspension was diluted to ~10^5^ CFU/ml, and 100 μl aliquots were added to sterile 96-well flat-bottom polystyrene plates containing 100 μl of CAMHB. The plates were incubated at 37°C for five hours to allow biofilm formation. Wells were then rinsed with 0.45% saline to remove planktonic cells and filled with 100 μl of CAMHB containing meropenem at 0.008–16 μg/ml combined with a fixed 8 μg/ml of vaborbactam. The plates were incubated for an additional 20 h at 37°C. Untreated wells served as growth controls. Following treatment, wells were washed twice with saline, and biofilms were mechanically disrupted by scraping. Cells were resuspended in 100 μl of saline, serially diluted, and plated on MacConkey agar for CFU enumeration. *K. pneumoniae* ATCC 13883 was included as a reference strain to standardize biofilm quantification procedures. The MBEC_90_ was defined as the lowest antibiotic concentration, resulting in a ≥90% reduction in viable biofilm-associated cells relative to the untreated control.

### 2.6 Phenotypic characterization of virulence-associated traits

Biofilm formation was assessed using a static microtiter plate assay. Strains were cultured in CAMHB at 37°C with agitation (150 rpm) for 24 hours, washed, adjusted to a 0.5 McFarland standard, and inoculated (100 μl) into 96-well flat-bottom polystyrene plates. Following 24 hours of static incubation at 37°C, wells were washed with phosphate-buffered saline (PBS), air-dried for 45 minutes, stained with 0.1% crystal violet (CV), and destained with ethanol-acetone, 4:1. Biofilm biomass was quantified by measuring absorbance at 570 nm (Sivori et al., 2024).

Planktonic growth was evaluated using a resazurin-based fluorescence assay. Bacterial suspensions (~1×10^5^ CFU/ml) were inoculated into 96-well plates containing CAMHB and 0.01% resazurin. Plates were incubated at 37°C, and fluorescence (excitation/emission: 560/590 nm) was measured every 30 minutes for 24 hours. Growth was expressed as the area under the fluorescence curve (AUC) relative to untreated controls.

Overnight bacterial cultures were harvested by centrifugation at 9,000 × g and resuspended in phosphate-buffered saline (PBS) to an optical density at 600 nm (OD_600_) of 1.0. Cell suspensions were centrifuged at 1,000 × g for 5 minutes to separate capsular material released into the supernatant. The OD_600_ of each supernatant was then measured and normalized to the initial OD_600_ of the corresponding bacterial suspension prior to centrifugation (Di Domenico et al., 2020).

Siderophore production was measured using the chrome azurol S (CAS) assay. Cultures (~1×10^5^ CFU/ml) were grown in CAMHB, centrifuged, and 100 μl of supernatant was mixed with 100 μl of CAS reagent in a 96-well plate. After 20 minutes at room temperature, absorbance was recorded at 630 nm. Siderophore activity was calculated as percent siderophore units (PSU) using the formula: PSU (%) = [(Ar − As)/Ar] × 100, where *Ar* is the absorbance of the reference (uninoculated control) and *As* the absorbance of the sample (Guembe et al., 2025).

The hypermucoviscosity phenotype was evaluated by the string test. A bacterial colony was touched with a standard inoculation loop and lifted vertically; a string length ≥5 mm was interpreted as positive. All assays were performed in biological triplicate (Di Domenico et al., 2020).

### 2.7 *In vivo* infection model using *Galleria mellonella*

The *Galleria mellonella* infection model was used to evaluate the *in vivo* efficacy of MEV against CRKP. Larvae (250–320 mg; Bigserpens, Paliano, Italy) were selected based on size and lack of melanization. Bacterial strains were cultured in CAMHB to mid-logarithmic phase (OD_600_ = 0.4–0.6), harvested by centrifugation, washed in PBS, and adjusted to a final concentration of ~1×10^5^ CFU per larva. Infection was established by injecting 10 μl of the bacterial suspension into the hemocoel via the last right proleg using a Hamilton syringe (10 μl volume, 26s gauge needle). Specifically, 30 minutes post-infection, treatment was administered by injecting 10 μl of MEV (Sigma–Aldrich) at 4 mg/kg. Control groups included untreated infected larvae and uninfected larvae injected with PBS. For each condition, 10 larvae were used. Infected larvae were incubated at 37°C in the dark, and their survival was monitored over 48 hours. Survival data were analyzed using the Mantel-Cox log-rank test (GraphPad Prism v10.0, GraphPad Software, San Diego, CA, USA). Experimental procedures were adapted from published protocols (Ignasiak and Maxwell, 2017; Guembe et al., 2025).

### 2.8 Statistical analysis

Statistical analysis was performed using GraphPad Prism v10.0 (GraphPad Software, San Diego, CA, USA) and IBM SPSS Statistics for Windows, Version 21.0 (IBM Corp., Armonk, NY, USA). Continuous variables are presented as mean ± standard deviation (SD) or as median with interquartile range (IQR), as appropriate. One-way analysis of variance (ANOVA) followed by Tukey’s multiple comparisons test was used to evaluate differences among groups for virulence-associated phenotypic traits. Kaplan–Meier survival curves for the *G. mellonella* model were analyzed using the log-rank (Mantel-Cox) test. A p-value < 0.05 was considered statistically significant for all comparisons. All experiments were conducted in biological triplicate unless otherwise stated.

## 3 Results

### 3.1 Isolation of carbapenem-resistant *K. pneumoniae* from catheter-related bloodstream infections

Two hospitalized patients in an orthopedic oncology unit were diagnosed with CRBSIs. The isolates were obtained from blood cultures collected via central venous access.

**Figure 1:**
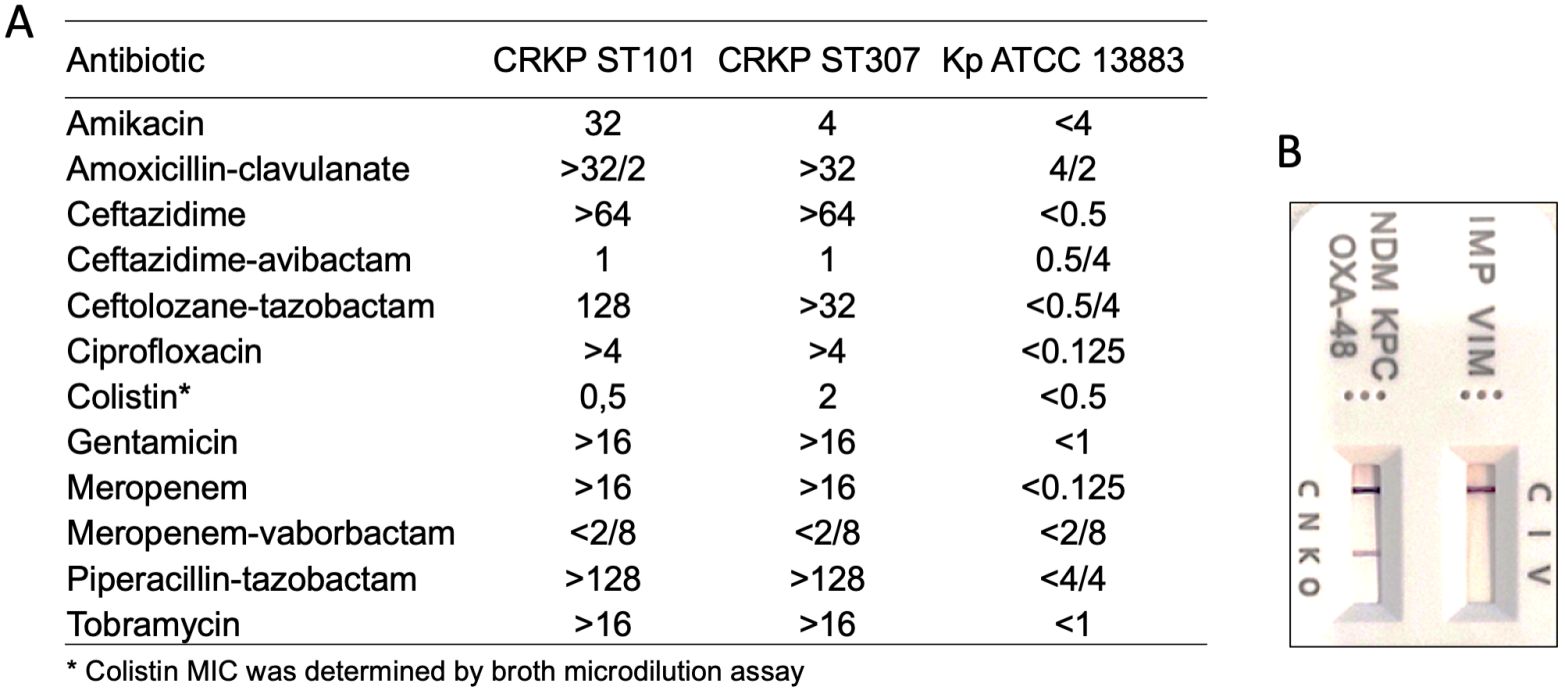
Antimicrobial susceptibility profiles and carbapenemase detection in *Klebsiella pneumoniae* strains. **(A)** Minimum inhibitory concentrations (MICs, μg/ml) for a panel of antibiotics were determined for CRKP ST101, CRKP ST307, and the reference strain *K*. *pneumoniae* ATCC 13883 using broth microdilution or automated systems, as appropriate. **(B)** Detection of carbapenemase enzymes was performed using a lateral flow immunoassay. Test bands indicate the presence of specific carbapenemases: IMP (I), VIM (V), NDM (N), KPC (K), and OXA-48-like enzymes (O). The control (C) band serves as an internal validity marker for test performance.

The antibiotic susceptibility testing of *K. pneumoniae* CRKP ST101 and CRKP ST307 was reported in **Figure 1A**. The *K. pneumoniae* ATCC 13883 (Kp ATCC 13883) was included as a reference strain (**Figure 1A**). CRKP ST101 and CRKP ST307 displayed extensive drug resistance, with high MICs to β-lactams, aminoglycosides, ciprofloxacin, and meropenem (MIC >16 μg/ml).

In contrast, KP ATCC 13883 remained susceptible to all tested agents, with low MIC values across all antibiotic classes. Notably, both CRKP ST101 and CRKP ST307 isolates were susceptible to MEV (MIC ≤ 2/8 μg/ml) despite their resistance to meropenem alone. The presence of carbapenemase enzymes was further verified by lateral flow immunoassay (**Figure 1B**). Colistin retained activity against CRKP ST101 (MIC = 0.5 μg/ml) but showed reduced efficacy against CRKP ST307 (MIC = 2 μg/ml), as determined by broth microdilution.

### 3.2 Genetic determinants and plasmid composition of two clinical CRKP isolates

**Figure 2:**
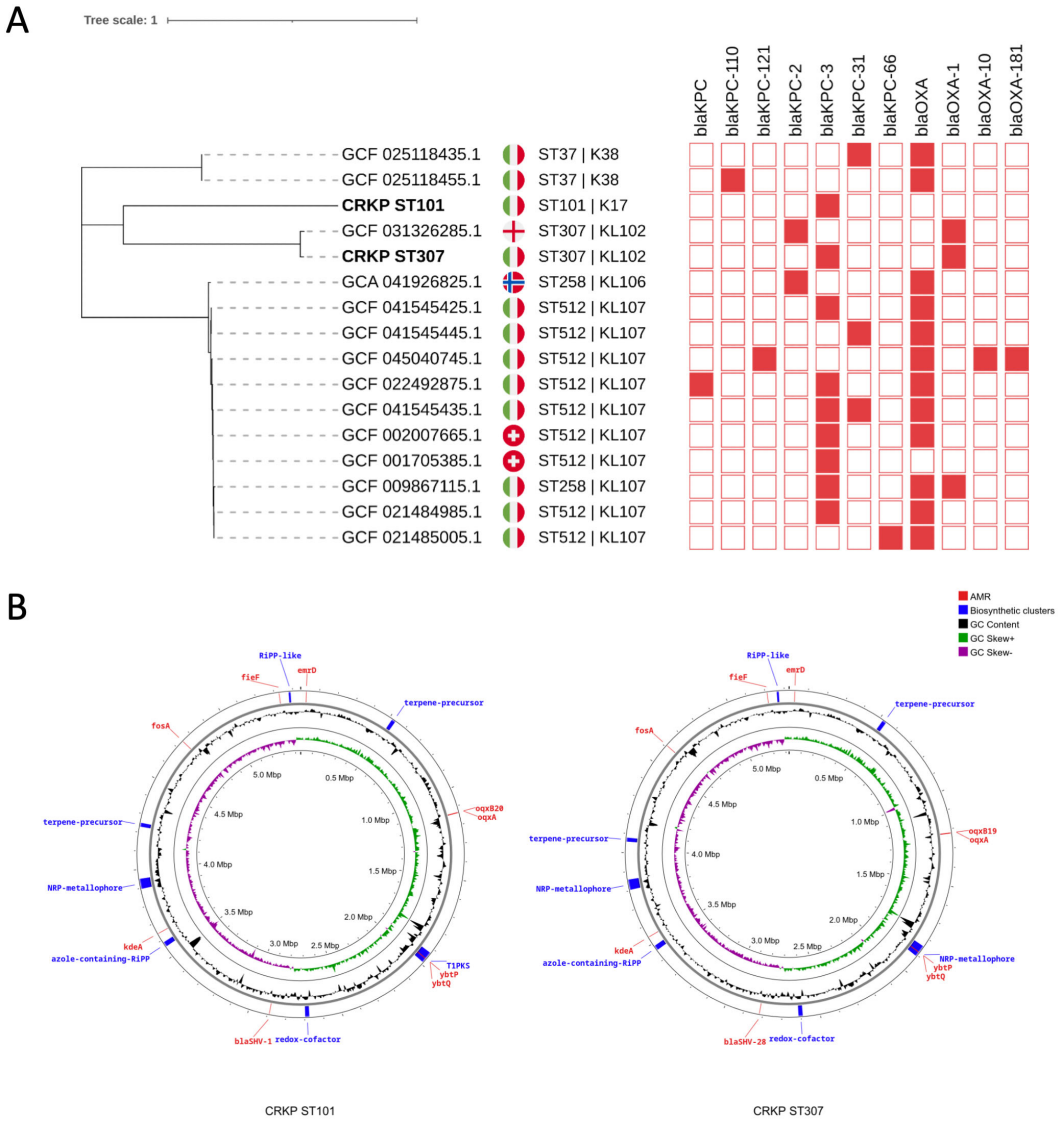
Phylogenetic analysis and genomic features of *K*. *pneumoniae* isolates. **(A)** A maximum-likelihood phylogenetic tree was constructed based on whole-genome sequences to assess the relatedness among *K*. *pneumoniae* strains. Isolates CRKP ST101 and CRKP ST307 (bold) are highlighted among reference genomes, revealing their phylogenetic placement. Genomes included in the analysis were filtered for high-quality completeness, and the presence of *bla*_KPC_ with metadata restricted to blood-derived isolates from European countries was confirmed. National flags indicate the country of origin. Sequence types (ST) and capsular loci (KL) are shown next to each genome. The presence or absence of *bla*_KPC_ or *bla*_OXA_ carbapenemase genes (red) are displayed in a binary heatmap. **(B)** Circular chromosomal maps of *Klebsiella pneumoniae* isolates CRKP ST101 and CRKP ST307. Chromosomal representations of CRKP ST101 (left) and CRKP ST307 (right) display annotated genomic features. Outer rings highlight predicted antimicrobial resistance (AMR) genes (red), biosynthetic gene clusters (blue), and relevant virulence or metabolic determinants. Inner rings display GC content (black), GC skew+ (green), and GC skew– (purple). Biosynthetic clusters include RIPP-like, nonribosomal peptide synthetase (NRP)-metallophore, azole-containing RIPP, and terpene precursor regions.

Whole-genome sequencing of the *K. pneumoniae* isolates identified two distinct multilocus sequence types (STs), ST101 and ST307 (**Figure 2A**), corresponding to capsule loci KL17 and KL102, respectively. The genome of the ST101 isolate (CRKP ST101) comprised a 5.44 Mb chromosome and seven plasmids ranging in size from 6,972 bp to 180,962 bp. The ST307 isolate (CRKP ST307) possessed a 5.32 Mb chromosome and two plasmids of 147,570 bp and 103,862 bp (**Figure 2B**). A maximum-likelihood phylogenetic tree was constructed based on core single-nucleotide polymorphisms (SNPs) from whole-genome sequences to investigate the relatedness of two CRKP isolates (CRKP ST101 and CRKP ST307) to reference genomes available in the NCBI Pathogens database. The inclusion criteria comprised genome assemblies of high quality or complete status, the presence of *bla*KPC, blood as the source of isolation, and origin from European countries. The analysis shows that both isolates cluster more closely with a strain from England than other Italian isolates (**Figure 2A**).

Virulence-associated genes typically linked to hypervirulent *K. pneumoniae* (hvKP), including *iucA*, *iroB*, *rmpA*, and *rmpA2*, were undetected in either isolate. CRKP ST101 and CRKP ST307 carried antimicrobial resistance determinants conferring resistance to three or more antibiotic classes (**Figure 2B**).

**Figure 3:**
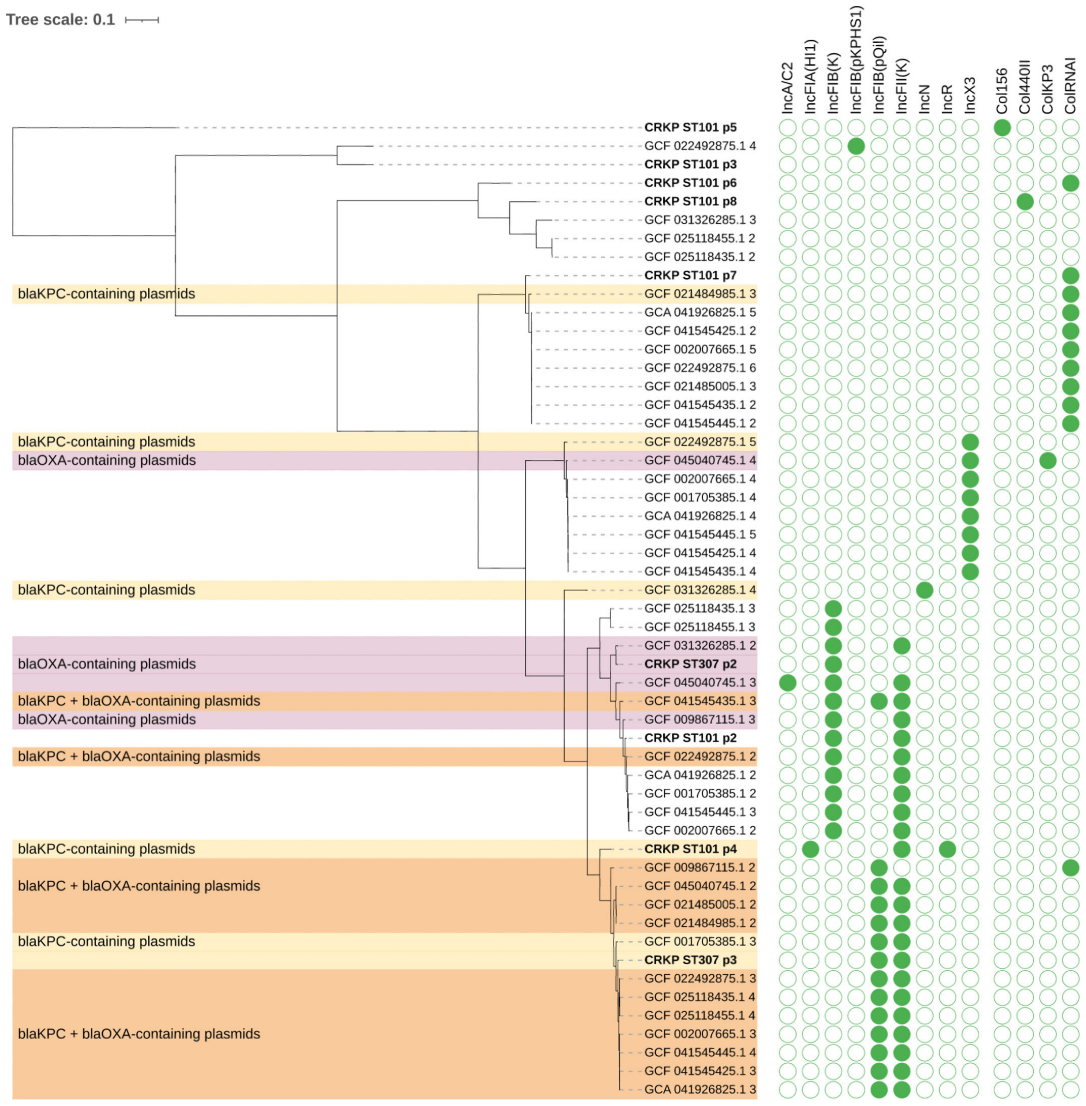
Phylogenetic tree of plasmids carrying carbapenemase genes *bla*_KPC_ and/or *bla*_OXA_ in *K. pneumoniae*. Plasmid sequences were clustered based on sequence similarity and colored according to the presence of *bla*_KPC_ (yellow), *bla*_OXA_ (purple), or both genes (orange). Plasmids (p) from CRKP ST101 and CRKP ST307 are highlighted (bold). The matrix on the right displays the presence (green) or absence (white) of plasmid replicons across plasmid genomes.

Plasmid phylogeny (**Figure 3**) revealed that the *bla*_KPC-3_ gene in CRKP ST101 was located on plasmid p4, which clustered with a group of *bla*_KPC_-carrying plasmids, including GCF_009867115.2, GCF_045040745.1, GCF_021848955.1, and GCF_001053585.1.3. These plasmids formed a distinct clade within the *bla*_KPC_-containing group and carried replicons associated with the IncFII incompatibility group. In CRKP ST307, plasmid p2 carried the *bla*_OXA-1_ gene and grouped with *bla*_OXA_-containing plasmids such as GCF_031362851.2 and GCF_045040745.1.3. This plasmid encoded an IncFIB replicon. Plasmid p3 harbored the *bla*_KPC-3_ gene and clustered with *bla*_KPC_-only plasmids, including GCF_001053585.1.3, and carried both IncFIB and IncFII replicons.

### 3.3 Phenotypic characterization of virulence-associated traits in CRKP isolates

**Figure 4:**
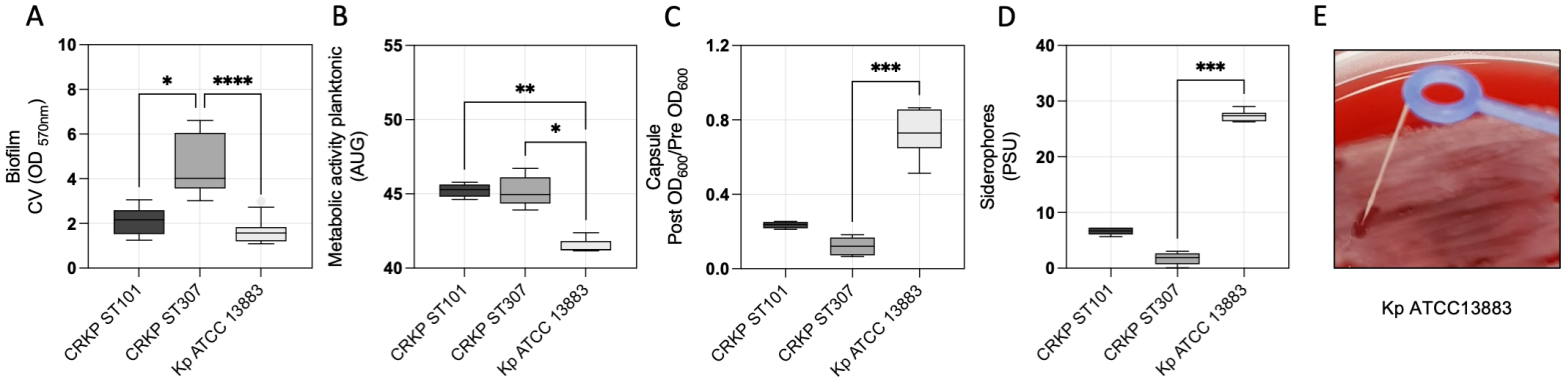
Virulence-associated traits in *K*. *pneumoniae* clinical isolates. **(A)** Quantitative analysis of biofilm biomass, **(B)** metabolic activity of planktonic cells, **(C)** capsule production, **(D)** and siderophore secretion in CRKP ST101, CRKP ST307, and Kp ATCC 13883. Statistical significance was determined using a one-way ANOVA followed by Tukey’s multiple comparisons test (p < 0.05). **(E)** A representative image of the string test performed on Kp ATCC 13883 to assess the hypermucoviscous phenotype. *P < 0.05; **P < 0.01; ***P < 0.001, ****P < 0.0001.

Biofilm formation, metabolic activity, capsule production, and siderophore secretion in CRKP ST101, CRKP ST307, and the reference strain Kp ATCC 13883 were analyzed to investigate the virulence-associated traits. CRKP ST307 exhibited significantly higher biofilm biomass than CRKP ST101 (p < 0.0292) and Kp ATCC 13883 (p < 0.0001) (**Figure 4A**). Metabolic activity, assessed using a resazurin-based assay, was lower in Kp ATCC 13883 compared to CRKP ST101 (p = 0.0074) and CRKP ST307 (p = 0.0148) (**Figure 4B**). Capsule production was significantly higher in Kp ATCC 13883 than in CRKP ST307 (p = 0.0002) (**Figure 4C**), while siderophore secretion was also greater in Kp ATCC 13883 (p = 0.0003) (**Figure 4D**). A positive string test identified Kp ATCC 13883 as the only strain exhibiting a hypermucoviscous (HMV) phenotype (**Figure 4E**).

### 3.4 Antibacterial Activity of MEV against planktonic and biofilm-associated CRKP Isolates

**Figure 5:**
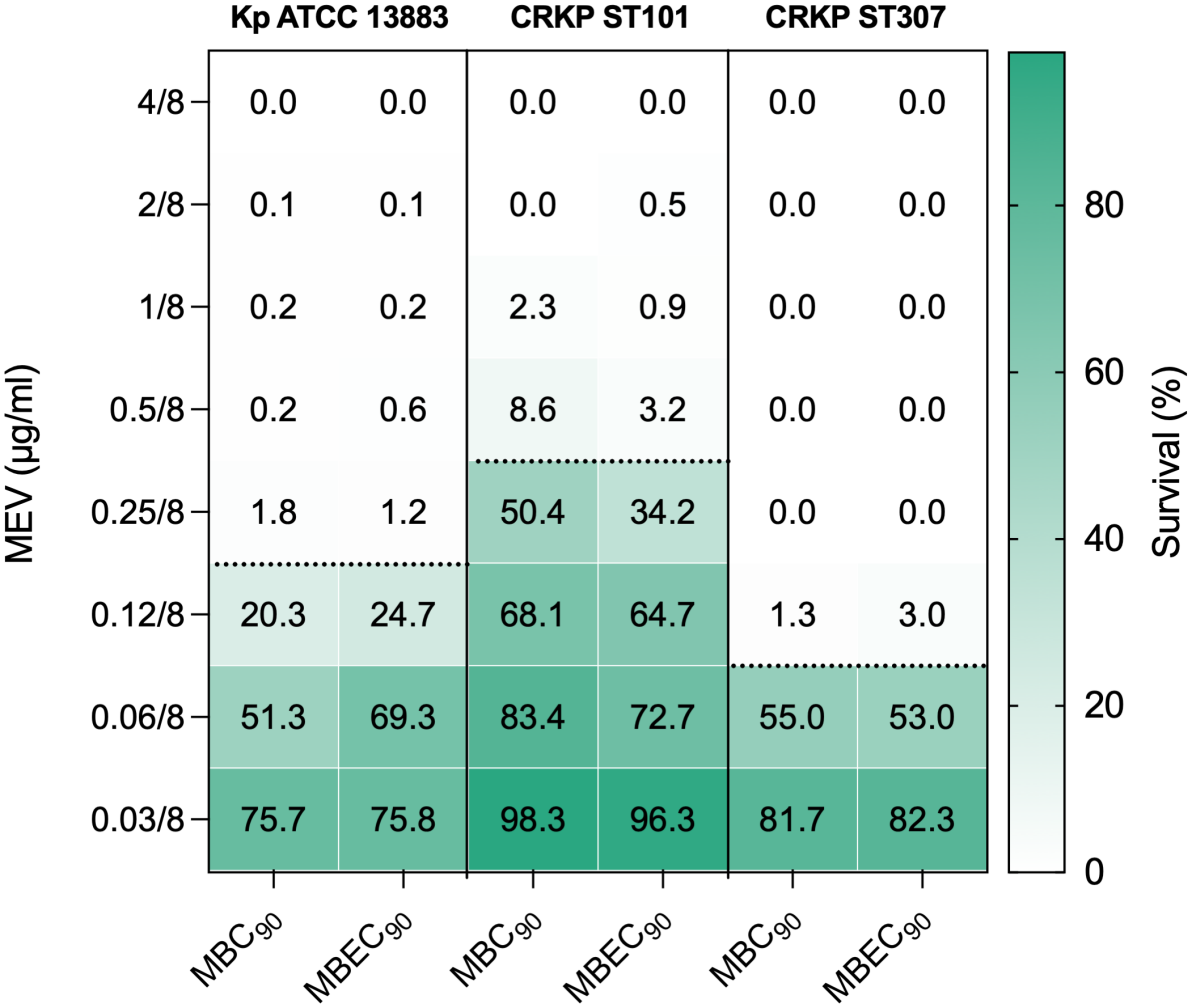
Antibacterial activity of MEV against *K. pneumoniae* planktonic and biofilm-associated cells. Bacterial survival was quantified following treatment with increasing concentrations of MEV and expressed as the percentage of CFU/ml relative to untreated controls. Data are shown for CRKP ST101, CRKP ST307, and the reference strain *K. pneumoniae* ATCC 13883 under planktonic and biofilm-associated conditions. The color gradient indicates growth compared to the untreated controls. The dashed black horizontal line indicates the 90% reduction threshold used to define the minimum bactericidal concentration (MBC_90_) for planktonic cells and the minimum biofilm eradication concentration (MBEC_90_) for biofilm-associated cells.

The antibacterial activity of MEV was assessed against *K. pneumoniae* isolates CRKP ST101, CRKP ST307, and the reference strain Kp ATCC 13883 under both planktonic and biofilm-associated conditions (**Figure 5**). Bacterial viability was determined following treatment with increasing concentrations of MEV, and survival was expressed as the percentage of CFU/ml relative to untreated controls. For planktonic cells, MEV induced a concentration-dependent reduction in bacterial survival across all tested strains. The minimum bactericidal concentration (MBC_90_), defined as the lowest concentration of MEV resulting in ≥90% reduction in CFU, was 0.5/8 μg/ml for CRKP ST101, 0.12/8 μg/ml for CRKP ST307, and 0.25/8 μg/ml for Kp ATCC 13883. Similar concentrations were required for biofilm-associated cells to achieve ≥90% reduction in viability. Minimum biofilm eradication concentration (MBEC_90_) values were 0.5/8 μg/ml for CRKP ST101, 0.12/8 μg/ml for CRKP ST307, and 0.25/8 μg/ml for the reference strain. MEV showed negligible activity at concentrations below 0.06/8 μg/ml across all strains and conditions.

### 3.5 *In vivo* assessment of virulence and therapeutic efficacy of MEV in the *Galleria mellonella* infection model

**Figure 6:**
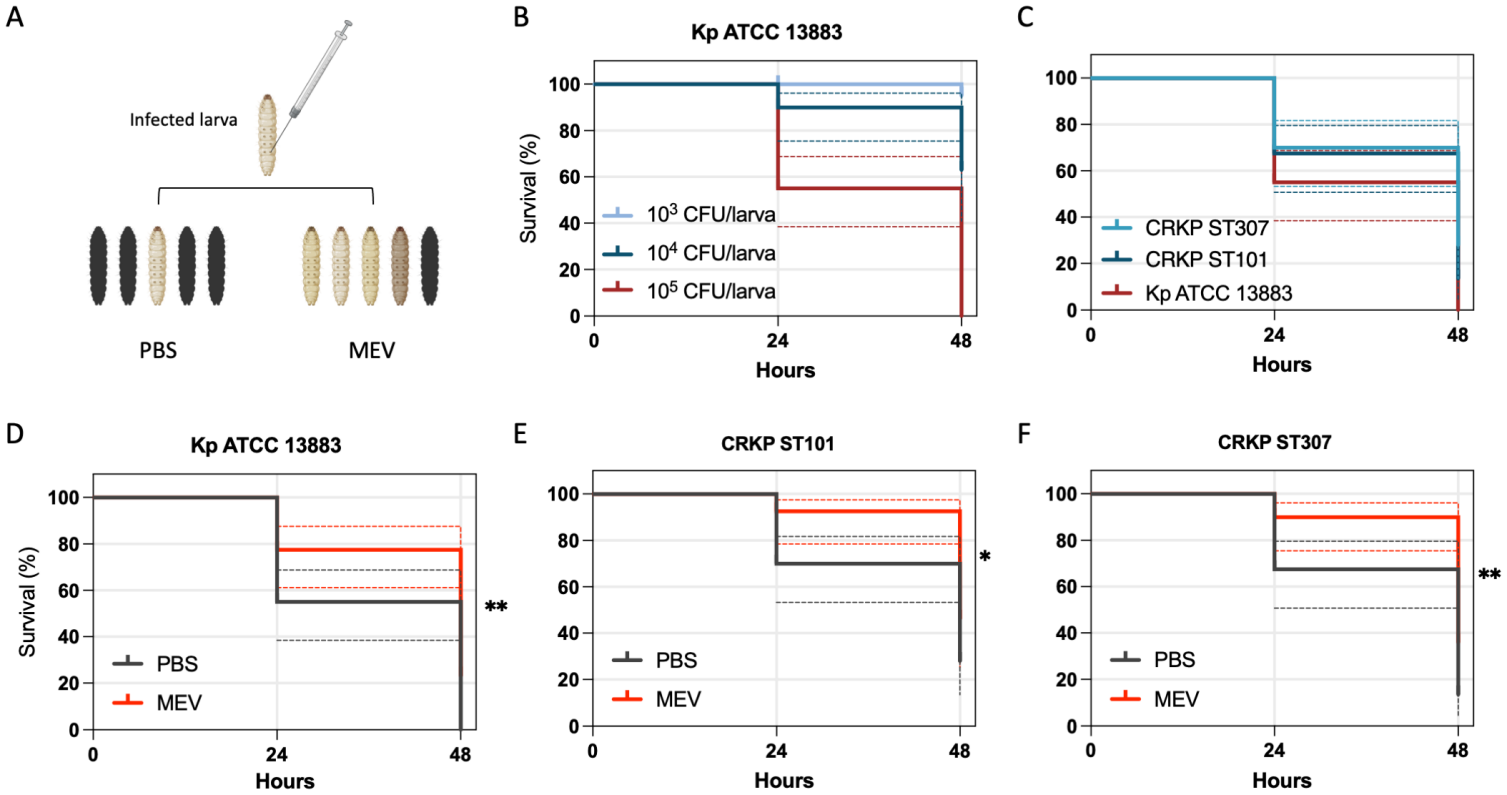
Kaplan-Meier survival curves of *G*. *mellonella* infected with *K*. *pneumoniae* and treated with MEV. **(A)** Schematic representation of the *G*. *mellonella* infection model, illustrating the injection procedure and representative images of healthy (light-colored) and dead (dark-colored) larvae. **(B)** Survival of *G*. *mellonella* following infection with Kp ATCC 13883 at inocula of 1×10³, 1×10^4^, and 1×10^5^ CFU/larva over 48 hours. **(C)** Comparative survival analysis of *G*. *mellonella* infected with Kp ATCC 13883, CRKP ST101, and CRKP ST307 at 1×10^5^ CFU/larva. **(D–F)** Kaplan-Meier survival curves of *G*. *mellonella* infected with Kp ATCC 13883 **(D)**, CRKP ST101 **(E)**, or CRKP ST307 **(F)** at 1×10^5^ CFU/larva and treated with a single dose of MEV (2g meropenem + 2g vaborbactam equivalent) administered 30 minutes post-infection. Control groups included larvae treated with either MEV (2 g/2 g equivalent) or PBS (Data not shown). Statistical significance was determined using the Log-rank test. *P < 0.05; **P < 0.01.

Survival assays in the *Galleria mellonella* infection model were conducted to evaluate the virulence of CRKP isolates and the *in vivo* efficacy of MEV (**Figure 6A**). A dose-dependent mortality was observed following infection with Kp ATCC 13883, with 100% lethality reached at an inoculum of 1 × 10^5^ CFU/larva within 24 hours (**Figure 6B**). This concentration was used to test the activity of MEV in the control Kp ATCC 13883, CRKP ST101, and CRKP ST307. Comparative analysis of larvae infected with 1×10^5^ CFU/larva revealed significantly increased mortality in larvae infected with Kp ATCC 13883 (p = 0.0052) compared to both CRKP ST101 and CRKP ST307 (**Figure 6C**). At 48 hours post-infection, survival rates were 40.0% for CRKP ST101, 20.0% for CRKP ST307, and 0% for Kp ATCC 13883. Treatment with MEV (2 g meropenem + 2 g vaborbactam equivalent), administered 30 minutes post-infection, significantly improved survival in infected larvae (**Figures 6D-F**). Larvae infected with *K. pneumoniae* and treated with a single dose of MEV exhibited a significantly higher survival rate compared to untreated Kp ATCC 13883 (p = 0.0019), CRKP ST101 (p = 0.0204), and CRKP ST307 (p = 0.0058), at 24 hours.

## 4 Discussion

The increasing prevalence of CRKP in oncology settings represents a critical clinical challenge, particularly in patients requiring prolonged use of indwelling devices. In this study, we characterized two CRKP isolates from CRBSIs in an orthopedic oncology unit, integrating genomic, phenotypic, and *in vivo* data to evaluate the activity of MEV. Whole-genome sequencing revealed that the isolates belonged to high-risk clones ST101 and ST307, both associated with extensive antimicrobial resistance (AMR) and nosocomial transmission. These isolates harbored clinically relevant resistance determinants, including *bla*_KPC-3_ and *bla*_OXA_, distributed across IncFII and IncFIB plasmid replicons. Both sequence types have been increasingly reported in bloodstream infections across Italy, with ST101 and ST307 representing emerging epidemic clones associated with hospital outbreaks and poor clinical outcomes in southern regions (Loconsole et al., 2020; Di Pilato et al., 2024). These isolates harbored clinically relevant resistance determinants, including *bla*_KPC-3_ and *bla*_OXA_. This observation aligns with national surveillance studies reporting KPC-3 as the most prevalent carbapenemase variant among *K. pneumoniae* isolates circulating in Italy (Giani et al., 2013; Bonura et al., 2015; Calia et al., 2017; Fasciana et al., 2019; Guembe et al., 2025). The identified genetic determinants are also consistent with previous reports describing the plasmid-mediated convergence of resistance in CRKP strains circulating in healthcare environments (Wyres et al., 2020; Di Pilato et al., 2024; Zhao et al., 2024). A recent nationwide genomic surveillance in Italy documented the widespread circulation of high-risk clones, particularly those within clonal complexes CC258, CC101, and CC307, among CRKP (Bianco et al., 2025). These lineages were also predominant among strains resistant to novel β-lactam/β-lactamase inhibitor combinations, including MEV and imipenem/relebactam and ceftazidime/avibactam. Although all isolates in our study remained susceptible to MEV, they belonged to clonal backgrounds (ST101 and ST307) that have been previously implicated in cross-resistance to multiple β-lactam/β-lactam inhibitor combinations. This overlap highlights the potential for further resistance development and supports the need for ongoing genomic monitoring to track the dissemination of epidemiologically successful clones and detect early signs of reduced susceptibility.

Phenotypic assays demonstrated that CRKP ST307 exhibited enhanced biofilm production and metabolic activity relative to CRKP ST101 and the reference strain Kp ATCC 13883 in the absence of hypervirulence-associated markers. These findings align with previous observations that classical CRKP lineages can express robust biofilm phenotypes contributing to persistence and therapeutic recalcitrance in the bloodstream and hospital-acquired infections (Gominet et al., 2017; Di Domenico et al., 2020; Fabrizio et al., 2024; Guerra et al., 2025). In contrast, biofilm production did not correlate with increased *in vivo* mortality in the *G. mellonella* model. Despite CRKP ST307 exhibiting the highest biofilm biomass *in vitro*, larvae infected with this strain demonstrated lower mortality rates than those infected with the reference strain Kp ATCC 13883. This dissociation between biofilm-forming capacity and acute virulence in *G. mellonella* suggests that biofilm-associated traits may primarily contribute to persistence and antibiotic tolerance rather than acute lethality in this model (Vuotto et al., 2017; Di Domenico et al., 2020). The observation supports previous studies indicating that while biofilm formation enhances survival under antimicrobial pressure and host immunity, it does not necessarily translate to increased pathogenicity in short-term infection models lacking complex immune responses (Wand et al., 2012; Benthall et al., 2015; Guembe et al., 2025).

The pronounced virulence of Kp ATCC 13883 in the *G. mellonella* infection model, as evidenced by complete lethality at 1×10^5^ CFU/larva within 24 hours, can be attributed to its elevated production of capsule and siderophores. Quantitative assays confirmed significantly greater capsule polysaccharide, HMV phenotype, and siderophore secretion in Kp ATCC 13883 compared to CRKP ST101 and CRKP ST307. Both factors have been independently associated with increased immune evasion and enhanced fitness in host environments, including invertebrate models (Paczosa and Mecsas, 2016). Capsule production inhibits phagocytosis and complement activation, while siderophores facilitate iron acquisition under host-imposed nutritional immunity, contributing synergistically to virulence (Paczosa and Mecsas, 2016). These findings are consistent with previous studies demonstrating that hypercapsulated and siderophore-rich *K. pneumoniae* strains elicit high mortality in *G. mellonella* independent of classical hypervirulence genotypes (Pu et al., 2023).

MEV exhibited potent bactericidal activity against both planktonic and biofilm-associated cells. MBC_90_ and MBEC_90_ values for CRKP ST101, CRKP ST307, and Kp ATCC 13883 were ≤0.5/8 μg/ml, confirming the efficacy of MEV across multiple growth states. This result is consistent with *in vitro* studies demonstrating MEV’s preserved activity against KPC-producing strains despite resistance to meropenem monotherapy (Tumbarello et al., 2024; Giuliano et al., 2025). The low MBEC_90_ values further support its potential use in device-associated infections where biofilm formation contributes to therapeutic failure.

In the *G. mellonella* model, MEV significantly improved survival in larvae infected with CRKP ST101 or CRKP ST307. These findings are in line with prior preclinical studies demonstrating the *in vivo* efficacy of MEV in bloodstream and device-related infection models (Tiseo et al., 2024). Notably, while both strains were susceptible to MEV *in vitro*, differential virulence was observed, with CRKP ST307 exhibiting higher biofilm biomass and lower capsule and siderophore production. This phenotypic variation may reflect strain-specific adaptations influencing pathogenesis and treatment response.

The *in vivo* efficacy of MEV observed in the *G. mellonella* model must be interpreted within the context of the experimental design, particularly regarding pharmacokinetics and dosage regimens. In clinical settings, MEV is administered as a combination of 2 g meropenem and 2 g vaborbactam every 8 hours by intravenous infusion over 3 hours. This extended infusion strategy ensures sustained plasma concentrations exceeding the MIC, which is critical for optimizing bactericidal activity against *K. pneumoniae*, particularly in severe infections such as bacteremia. In contrast, the *G. mellonella* model employed a single-dose administration of MEV (2 g meropenem + 2 g vaborbactam equivalent) delivered 30 minutes post-infection. While this regimen enabled a comparative assessment of strain-specific virulence and drug responsiveness, it does not replicate the pharmacodynamic exposure achieved in human therapy. This discrepancy highlights a key limitation of the study, as the simplified dosing in the invertebrate model does not capture the complexity of drug absorption, distribution, metabolism, and elimination in mammalian systems. Additionally, although *G. mellonella* offers a cost-effective model for the preliminary evaluation of antimicrobial efficacy and virulence, it lacks the immunological and physiological complexity of vertebrate hosts.

Further limitations include the small number of isolates examined, which restricts the generalizability of our findings. While our two CRKP strains provide valuable mechanistic insights into MEV activity, they may not fully represent the genetic and phenotypic diversity observed among CRKP in clinical settings. For instance, Giuliano et al. (2025) described two KPC-producing CRKP isolates causing ventriculitis, which responded favorably to MEV therapy, highlighting the utility of such studies despite the inherent limitations of small sample sizes. Similarly, Larcher et al. (2023) reported a single case of bloodstream infection due to a KPC-3-producing *K. pneumoniae* in a critically ill patient with augmented renal clearance. The successful use of continuous infusion MEV in this context illustrates how individualized therapeutic strategies can be informed by detailed isolate characterization even in single-isolate studies. These examples underscore the need for broader datasets to capture the full spectrum of resistance mechanisms, virulence factors, and biofilm phenotypes within CRKP populations. Moreover, the absence of pharmacokinetic/pharmacodynamic analyses in our study limits the translation of *in vitro* findings into optimized dosing regimens for clinical use. Future studies incorporating dynamic infection models and standard human-equivalent dosing schedules will be crucial for more accurately assessing the translational potential of MEV in treating CRKP-associated bloodstream infections.

Furthermore, caution is warranted in interpreting MEV’s activity against biofilm-associated *K. pneumoniae*. Although MEV showed *in vitro* activity against mature biofilms, the extracellular matrix and reduced metabolic activity of biofilm-embedded cells may limit antibiotic penetration and bactericidal efficacy *in vivo*. These features may limit the clinical utility of *in vitro* MBEC_90_ values as predictors of treatment success in device-associated infections.

Collectively, our findings highlight the genomic complexity and phenotypic heterogeneity of CRKP strains causing CRBSI in immunocompromised hosts. MEV demonstrated activity against planktonic and biofilm-associated CRKP and improved survival in an invertebrate infection model. These data support the continued evaluation of MEV as a therapeutic option for CRKP-associated bloodstream infections and underscore the importance of integrating genomic surveillance with functional assays to inform antimicrobial stewardship strategies in high-risk populations.

## Data Availability

The datasets presented in this study can be found in online repositories. The names of the repository/repositories and accession number(s) can be found below: https://www.ebi.ac.uk/ena, PRJEB88190.
